# RAD sequencing reveals genomewide divergence between independent invasions of the European green crab (*Carcinus maenas*) in the Northwest Atlantic

**DOI:** 10.1002/ece3.2872

**Published:** 2017-03-14

**Authors:** Nicholas W. Jeffery, Claudio DiBacco, Mallory Van Wyngaarden, Lorraine C. Hamilton, Ryan R. E. Stanley, Renée Bernier, Jennifer FitzGerald, K. Matheson, C. H. McKenzie, Praveen Nadukkalam Ravindran, Robert Beiko, Ian R. Bradbury

**Affiliations:** ^1^ Northwest Atlantic Fisheries CentreFisheries and Oceans CanadaSt. John'sNLCanada; ^2^ Bedford Institute of OceanographyFisheries and Oceans CanadaDartmouthNSCanada; ^3^ Ocean Sciences Center and Biology DepartmentMemorial University of NewfoundlandSt John'sNLCanada; ^4^ Aquatic Biotechnology LaboratoryBedford Institute of OceanographyDartmouthNova ScotiaCanada; ^5^ Gulf Fisheries CentreFisheries and Oceans CanadaMonctonNew BrunswickCanada; ^6^ Faculty of Computer ScienceDalhousie UniversityHalifaxNova ScotiaCanada

**Keywords:** *Carcinus maenas*, COI, European green crab, population structure, restriction‐site‐associated DNA sequencing

## Abstract

Genomic studies of invasive species can reveal both invasive pathways and functional differences underpinning patterns of colonization success. The European green crab (*Carcinus maenas*) was initially introduced to eastern North America nearly 200 years ago where it expanded northwards to eastern Nova Scotia. A subsequent invasion to Nova Scotia from a northern European source allowed further range expansion, providing a unique opportunity to study the invasion genomics of a species with multiple invasions. Here, we use restriction‐site‐associated DNA sequencing‐derived SNPs to explore fine‐scale genomewide differentiation between these two invasions. We identified 9137 loci from green crab sampled from 11 locations along eastern North America and compared spatial variation to mitochondrial COI sequence variation used previously to characterize these invasions. Overall spatial divergence among invasions was high (pairwise *F*_ST_ ~0.001 to 0.15) and spread across many loci, with a mean *F*_ST_ ~0.052 and 52% of loci examined characterized by *F*_ST_ values >0.05. The majority of the most divergent loci (i.e., outliers, ~1.2%) displayed latitudinal clines in allele frequency highlighting extensive genomic divergence among the invasions. Discriminant analysis of principal components (both neutral and outlier loci) clearly resolved the two invasions spatially and was highly correlated with mitochondrial divergence. Our results reveal extensive cryptic intraspecific genomic diversity associated with differing patterns of colonization success and demonstrates clear utility for genomic approaches to delineating the distribution and colonization success of aquatic invasive species.

## 1 Introduction

Global invasions of plants and animals are occurring at an increasing rate accelerated by human impacts and environmental change (Levings, Kieser, Jamieson, & Dudas, 2002; Mooney & Cleland, 2001; Ricciardi, 2006). Invasions can have significant ecological and evolutionary consequences at the species (McDonald, Parchman, Bower, Hubert, & Rahel, 2008), community (Matheson et al., 2016; Robinson & Dickerson, 1987), and ecosystem levels (Hänel & Chown, 1998). Ecological impacts have been well studied in coastal marine habitats, which represent some of the most heavily invaded ecosystems due to human‐mediated introductions from ship ballast and hull fouling, as well as the aquarium trade and aquaculture activities (Grosholz, 2002). Ecological impacts of aquatic invaders range from large‐scale reductions in native biodiversity (Molnar, Gamboa, Revenga, & Spalding, 2008) to the local destruction of marine habitats such as eelgrass or shellfish beds (Garbary, Miller, Williams, & Seymour, 2014; Matheson et al., 2016; McClenachan, O'Connor, & Reynolds, 2015), which can include commercially important species (Floyd & Williams, 2004). In contrast to ecological impacts, evolutionary dynamics of marine invasions are less well studied including both the genomic basis for invasiveness (Lee, 2002) and evolutionary consequences of invasion (Chown et al., 2015; Cristescu, 2015). Increasingly, it is becoming apparent that cryptic diversity can contribute to variation in invasion success and ultimately to impacts on marine and freshwater ecosystems (e.g., Folino‐Rorem, Darling, & D'Ausilio, 2009; Lee, 1999; Sherman et al., 2016). A better understanding of the presence of intraspecific diversity within invasive species and how it correlates with variation in invasion success is necessary to successfully manage invaders and mitigate ecological and evolutionary impacts.

An evolutionary perspective of marine invasion dynamics and of cryptic diversity within invaders has been limited by the huge interspecific diversity among invasive taxa and a lack of genomic resources. The ability to study “invasion genomics” using relatively new tools such as restriction‐site‐associated DNA sequencing (RAD‐seq), RNA‐seq genotyping, or transcriptomics allows the exploration of evolutionary history, connectivity, range expansion rates, and invasion patterns with markers from across the entire genome without the requirement of an existing whole‐genome sequence (Chown et al., 2015; Rius & Darling, 2014; Sherman et al., 2016; Viard, David, & Darling, 2016). Such genomewide sampling techniques can be used to identify so‐called invasion genes or marker associations with environmental factors (Chown et al., 2015; Wang et al., 2011) and can be useful in understanding patterns and rates of expansion (e.g., Krehenwinkel & Tautz, 2013), as well as the roles of neutral and adaptive pressures in invasion dynamics (e.g., Tepolt & Palumbi, 2015). Genomewide markers are particularly useful for marine invasive species whose adult and/or larval phases may be transported by coastal currents or by human‐mediated vectors, and thus, invasion genomics can greatly facilitate our understanding of the processes behind the initial invasion and subsequent impacts at a scale not attained with typical genetic markers.

European green crab (*Carcinus maenas*) are a notorious invasive species now present on every continent with the exception of Antarctica (Roman, 2006). Green crab have a prolonged larval phase and high tolerance of variable environmental conditions allowing for further expansion throughout their introduced ranges, including both the Pacific and Atlantic coasts of North America (Roman, 2006). In the northwest Atlantic, green crab likely experienced multiple cryptic introductions, having invaded the eastern USA and Canada at least twice from genetically distinct northern and southern European populations in 1817 and again in the 1980s (Darling, Tsai, Blakeslee, & Roman, 2014; Roman, 2006). This species’ range is continuing to expand, having recently been discovered in Placentia Bay, Newfoundland, in 2007, with a likely introduction *c*. 2002 from a Scotian Shelf population (Blakeslee et al., 2010). Green crab population structure has been investigated previously using traditional genetic markers, including mitochondrial DNA (16S, Geller, Walton, Grosholz, & Ruiz, 1997; COI, Darling, Bagley, Roman, Tepolt, & Geller, 2008; Roman & Palumbi, 2004; Roman, 2006) and microsatellites (Darling, 2011; Darling et al., 2008, 2014; Pascoal et al., 2009; Tepolt, Bagley, Geller, & Blum 2006). In their native range, green crab show distinct broadscale structuring of island populations (i.e., Faeroe Islands and Iceland) and northern and southern European populations (Roman & Palumbi, 2004) using COI sequence data and fine‐scale (~450 km) structuring has been reported using microsatellite loci (Pascoal et al., 2009). In North America, spatial structuring is driven by both range expansion (Pringle, Blakeslee, Byers, & Roman, 2011) and secondary contact among invasions (Blakeslee et al., 2010; Darling et al., 2014; Tepolt & Palumbi, 2015). However, a genomewide comparison (i.e., RAD‐seq) of fine‐scale spatial structuring on the east coast has been absent and is warranted to compare these independent invasions and characterize the full genetic diversity of populations along this part of their invasive range.

Our overall objective was to evaluate population structuring using genomewide SNPs obtained by RAD‐seq for green crab from 11 locations in eastern North America to explore the genomic extent of differences between colonization events. Specifically, our goals were to (i) quantify genomewide differentiation among samples encompassing both the invasions in eastern North America from northern and southern European source populations, (ii) describe the spatial distribution of these two invasions using a large number of genomewide SNPs, and (iii) compare spatial variation at both highly divergent and selectively neutral SNPs, as well as the mitochondrial cytochrome oxidase I (COI) gene to better understand the evolutionary dynamics associated with the recent invasion and northwards expansion of the species. We predict (i) widespread genomic differentiation among these independent invasions based on existing work and the geographic isolation of the source populations and (ii) that larger numbers of loci used here will reveal finer‐scale population structure than has previously detected. We build directly on previous studies (e.g., Blakeslee et al., 2010; Darling et al., 2008, 2014; Tepolt & Palumbi, 2015) by comparing neutral and divergent SNPs to COI sequence data between the two invasions. The datasets developed here will allow for monitoring future range expansion of populations at both the southern and northern limits of the current range and also permit the formulation of conservation strategies that are region‐ and population‐specific.

## 2 Materials and Methods

### 2.1 Specimen collection and DNA extraction

**Figure 1:**
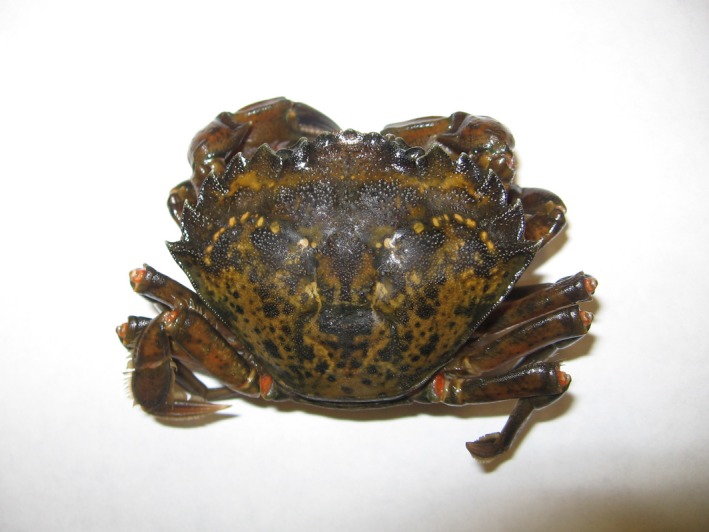
The green crab (*Carcinus maenas*) is native to Europe and has invaded eastern North America at least twice independently over the past 200 years. The range of this species has since increased both northwards and southwards, being found from Virginia to southern Newfoundland

**Figure 2:**
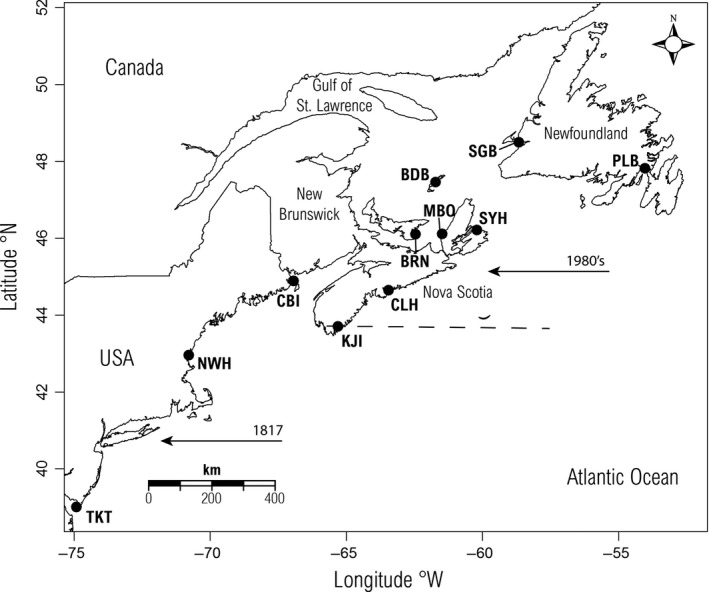
Map of eastern North America showing the 11 green crab sampling locations in the USA and Canada. The arrows represent the independent invasions of green crab into North America, and the dashed line indicates the approximate northern limit of the initial southern invasion to the New York area (1817) prior to the recent introduction of green crab to the Halifax region (1980s) (Audet et al., 2003)

Specimens were collected from 11 locations in eastern Canada and northeastern USA (Table 1, Figures 1 and 2). All samples were collected in 2011 with the exception of New Hampshire, which was sampled in 2013. Tissue samples were preserved in AllProtect (Qiagen, Toronto, ON, Canada) or 80% ethanol. DNA was isolated from the tissue samples using phenol: chloroform extraction or NucleoMag 96 Tissue (Macherey‐Nagel, Bethlehem, PA, USA) following the manufacturer's protocol, including RNase A (Qiagen) treatment. All DNA samples were quantified using the Qubit dsDNA HS Assay Kit (Life Technologies, Burlington, ON, Canada) with assays read on a Qubit v2.0 (Life Technologies) or the Quant‐iT PicoGreen dsDNA Assay Kit (Life Technologies) with assays read on a FLUOStar OPTIMA fluorescence plate reader (BMG Labtech, Ortenberg, Germany).

**Table 1:** List of sampling locations, including latitude and longitude, for green crab obtained for this study. The location code is provided, along with the number of individuals per location for which we obtained a full SNP panel and COI sequence. All locations were sampled in 2011, with the exception of NWH which was sampled in 2013

Location name	Location code	Latitude	Longitude	nSNPs	nCOI
St. George's Bay, NL	SGB	48.492	−58.658	22	22
Placentia Bay, NL	PLB	47.102	−54.184	22	36
Baie de Bassin, QC	BDB	47.473	−61.738	22	22
Sydney Harbour, NS	SYH	46.141	−60.202	22	22
Mabou, NS	MBO	46.069	−61.391	22	22
Brudenell River, PE	BRN	46.192	−62.588	22	22
Cole Harbour, NS	CLH	44.658	−63.442	22	23
Kejimkujik, NS	KJI	43.840	−64.805	21	27
Campobello Island, NB	CBI	44.888	−66.918	22	22
New Hampshire	NWH	43.04	−70.71	22	21
Tuckerton, NJ	TKT	39.588	−74.288	22	21

### 2.2 mtDNA analysis

A 502‐base pair fragment of the mitochondrial cytochrome *c* oxidase subunit I (COI) gene was amplified using M13F (‐21)/M13R (‐27) tagged version of the primers described in Roman and Palumbi (2004) (M13F‐CmaCOIF 5′ TGTAAAACGACGGCCAGTGCTTGAGCTGGCATAGTAGG 3′ and M13R‐CmaCOIR 5′ CAGGAAACAGCTATGACGAATGAGGTGTTTAGATTTCG 3′) (Integrated DNA Technologies, Coralville, IA, USA). PCRs were carried out in 50 μl volumes comprised of 100 ng DNA, 1× AmpliTaq 360 Buffer (Life Technologies), 1.5 mm MgCl_2_, 0.2 mm each nucleotide (dNTP), 0.5 μm each primer, and 0.025 U AmpliTaq 360 (Life Technologies). Thermal cycling conditions consisted of 94°C for 3 min; 30 cycles of 94°C for 30 s, 50°C for 30 s, and 72°C for 30 s; 1 cycle of 72°C for 10 min. To verify amplification, 5 μl of the PCR product was electrophoresed on a 1% agarose gel, visualized using SYBR Safe (Life Technologies) and documented using a Gel Logic 200 (Kodak). Forty microliters of the remaining PCR product was purified using QIAquick PCR purification kit (Qiagen). The purified PCR products were quantified as described above and sequenced in both directions using M13F (‐21) and M13R (‐27) and BigDye Terminator v3.1 cycle sequencing kit (Life Technologies) per the manufacturer's instructions with the following modification, and 0.5 μl of the BigDye Terminator v3.1 Ready Reaction Mix (Life Technologies) and 3.8 μl of the 5× sequencing buffer (Life Technologies) was used per 20 μl reaction. Sequencing products were separated by size on a 36‐cm array containing POP7 polymer on an AB3130xl genetic analyzer (Life Technologies). The sequencing results from both directions were assembled using Vector NTI Advance v11 (Life Technologies). For identification, the resulting sequence for each individual was then aligned to reference sequences, haplotypes H1–H6 and H8–H10 from Darling et al. (2008) (Genbank accession numbers FJ159008–FJ15016) using Vector NTI Advance v11 (Life Technologies).

### 2.2 RAD sequencing

RAD‐seq libraries were prepared as described by Etter, Preston, Bassham, Cresko, and Johnson (2011b; see also Etter, Bassham, Hohenlohe, Johnson, & Cresko, 2011a with modifications). All samples were normalized to 25 ng/μl. The DNA quality for all samples was verified by agarose gel electrophoresis of 100 ng of extracted DNA on a 0.7% agarose gel (DNA was visualized and documented as described above). Only DNA samples with a visible high molecular weight band on the agarose gel and that were positive for an M13F‐CmaCOIF/M13R‐CmaCOIR amplification product (see above) were used for library preparation. Each library was comprised of DNA samples from 22 individuals (1 μg DNA per individual) from the same geographic location with each individual sample having a different in‐line barcode in the P1 adapter. The P1 adapter in‐line barcodes ranged from 5 to 9 bp in length, and the sequences were chosen to ensure equal distribution of all nucleotides at each base position (including those that overlap with the restriction site) and maximizing the edit distance (Faircloth & Glenn, 2012). Based on edittags analysis (Faircloth & Glenn, 2012), the variable‐length barcodes had an edit distance ranging from 2 to 8 and a modal edit distance of 6. Gel size selection performed after sonication and PCR amplification was done on a Pippin Prep (Sage Science, Beverly, MA, USA) using the 2% agarose gel cassette (Sage Science) and size selection range of 300–500 bp. PCR amplification was done using Q5 Hot Start Master Mix (NEB, Whitby, ON, Canada) for all libraries. Amplification cycles for all libraries were 98°C for 30 s; 14 cycles of 98°C for 30 s, 65°C for 30 s, 72°C for 30 s; 1 cycle of 72°C for 5 min. All libraries were sequenced on a HiSeq 2000 (Illumina) as 100‐bp paired end sequences with one library per lane. Sequencing was performed at McGill University and Génome Québec Innovation Centre, Montréal, Canada.

### 2.3 Bioinformatics

RAD data for 242 individuals were cleaned and demultiplexed using *process_radtags* in Stacks v.1.21 (Catchen, Hohenlohe, Bassham, Amores, & Cresko, 2013). We used *ustacks* for de novo loci formation with a minimum depth of 5 to create a stack and a maximum distance of 4 between stacks. One individual from Kejimkujik (KJI) was removed due to low sequence coverage, and so, we retained 241 individuals overall. We then used *cstacks* to construct a locus catalog based on sequence identity with 1 mismatch allowed between stacks. We used settings of *M* = 4 as the maximum distance between two loci to merge and *n* = 1 to allow two catalog loci to merge in case they are differently fixed versions of the same locus. All RAD tags were 80 bp in length. These were then filtered in the Stacks *populations* module to include only RAD tags present in each population and in ≥75% of individuals and a minor allele frequency (MAF) greater than 5%. This dataset was filtered for missing data and deviations from Hardy–Weinberg equilibrium using PLINK (Purcell et al., 2007), where individuals missing >25% of loci and loci missing >5% of genotypes were removed. Finally, only one SNP per RAD tag was retained to remove the effects of physical linkage. The resulting panel was converted for subsequent analyses using PGDSpider v2.0.8.3 (Lischer & Excoffier, 2012) and the R package genepopedit (Stanley, Jeffery, Wringe, DiBacco, & Bradbury, 2017).

### 2.4 Population statistics and detecting divergent loci

**Figure 3:**
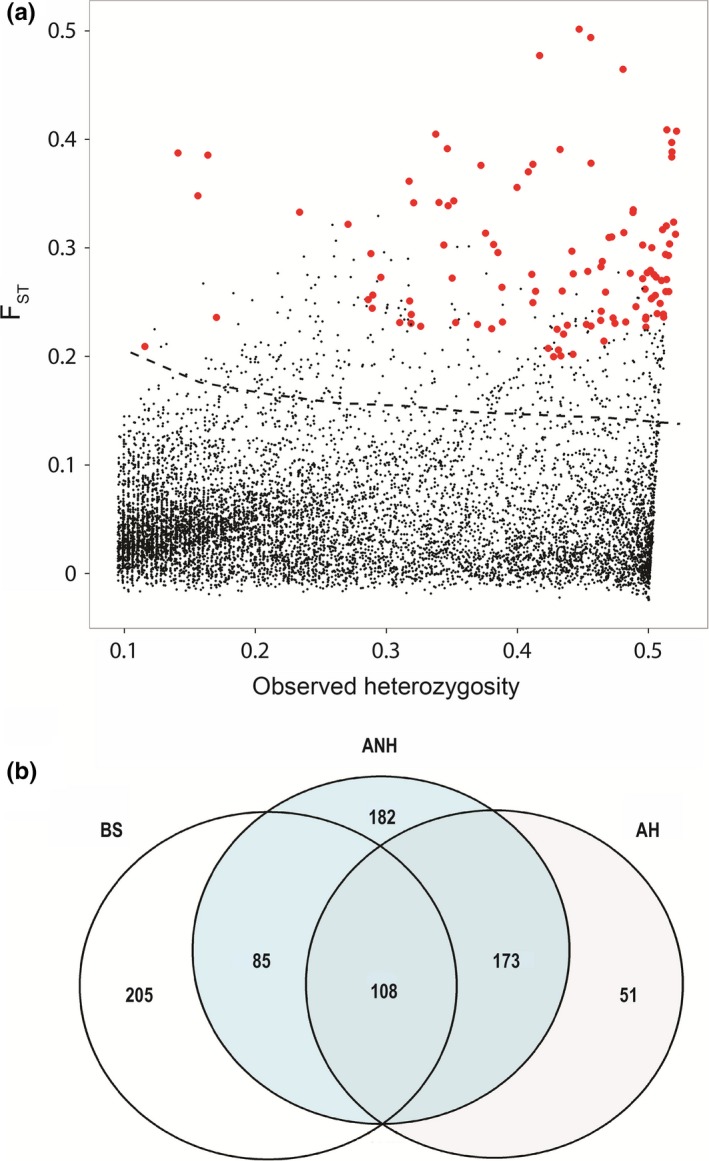
Outliers detected in the current study. (a) Locus‐specific *F*_ST_ values against heterozygosity from the nonhierarchical island model implemented in Arlequin. Loci common to all three detection methods are colored in red. The dashed line indicates the 1% quantile for outlier detection which contains all 108 outlier loci. (b) The overlap of outlier loci among BayeScan (BS) and Arlequin's nonhierarchical (ANH) and hierarchical (AH) island models. Note that the area of overlap between methods is not proportional to the number of SNPs in common

Multiple approaches were used to identify loci putatively experiencing adaptive selection (outliers). We first employed BayeScan v2.1 (Foll & Gaggiotti, 2008) with 50,000 burn‐in and 100,000 iterations, with prior odds set to 10. Second, we used a nonhierarchical island model in Arlequin v.3.5.2.1 (Excoffier & Lischer, 2010) to detect outlier loci using 200,000 simulations and 100 demes simulated with default expected heterozygosity settings. Third, we used the hierarchical island model in Arlequin with the same settings as the nonhierarchical model and 10 simulated groups to further test for outlier loci. While these methods have been shown to produce higher numbers of false positive outliers in some demographic scenarios, they also show increased power and perform similarly to other methods such as FLK (Bonhomme et al., 2010) under recent demographic expansion models (Lotterhos & Whitlock, 2014). Only loci identified as outliers by all three methods were considered to reduce the number of false positives, and our putatively neutral panel was comprised only of SNPs that were not detected as outliers by any of these methods (Figure 3). Outlier loci were subset from the full RAD panel using the *subset_genepop* function in genepopedit (Stanley et al., 2017). Observed (*H*_o_) and expected (*H*_e_) heterozygosities were calculated in the package hierfstat (Goudet, 2014) in R (R Core Team 2015) for each population within each SNP panel, as well as the average *H*_o_ and *H*_e_ for each panel. Locus‐specific heterozygosities and *F*_ST_ values were computed in hierfstat and diveRsity (Keenan, McGinnity, Cross, Crozier, & Prodöhl, 2013), respectively, with 1,000 bootstrap replicates. Heatmaps of linkage disequilibrium *r* ^2^ values and standardized allele frequencies, where the highest major allele frequency per SNP among populations is given a value of 1.0 and the lowest major allele frequency a value of 0, for outlier and neutral SNPs, were created using custom R scripts.

### 2.5 Population structure

Spatial structuring in neutral and outlier SNPs was explored using several methods. We conducted a discriminant analysis of principal components (DAPC) using adegenet 2.0.0 (Jombart, 2008; Jombart & Ahmed, 2011) in R to first find the number of clusters to best represent our 11 populations using the *find.clusters* function. All principal components were retained at this step. This function defines the best number of clusters using a Bayesian information criterion (BIC). We then conducted the DAPC using the defined clusters as prior information and retained the first 50 principal components to determine the best value of *K* for our data. The DAPC was conducted on the full SNP panel, as well as on the neutral and outlier datasets separately. Second, we used principal components analysis on the outlier and neutral SNP datasets to account for intrapopulation variance and compare with the results of our DAPC. Finally, Cavalli‐Sforza and Edwards’ (1967) chord distances were calculated from each panel of SNP data in POPULATIONS 1.2.33 (Langella, 2012). Neighbor‐joining (NJ) trees based on chord distances were constructed with 1,000 bootstrap replicates for all three SNP panels, and these were midpoint‐rooted and visualized in FigTree v1.4 (http://tree.bio.ed.ac.uk/software/figtree/). Geographic visualizations of *F*_ST_ similarities were built using GenGIS version 2.5 (PMID 23922841). Maps used in GenGIS were obtained from the Natural Earth project (http://www.naturalearthdata.com).

Spatial structuring in the SNP and COI datasets were also directly compared. We used Arlequin v3.5.2 (Excoffier & Lischer, 2010) to calculate pairwise *F*_ST_ values between each sampling location for the COI data and each of the three SNP panels. We then used a Mantel test with 1,000 permutations in the R package ade4 (Dray & Dufour, 2007) to compare pairwise *F*_ST_ values between the COI data and the full SNP panel. Additionally, we conducted a Mantel test with 1,000 permutations on linearized pairwise *F*_ST_ (Slatkin, 1995) values and geographic distance in ade4 for the full SNP panel and the COI dataset as a test of isolation by distance. Least‐cost distances between each pair of sampling locations were estimated in marmap (Pante & Benoit, 2013) implemented in R. An analysis of molecular variance (AMOVA) with 1,000 permutations was conducted for each dataset in Arlequin based on our optimal *K* = 2, where each sampled population was considered part of either a northern or southern group.

## 3 Results

Following initial filtering in Stacks, we obtained a catalog of 46,067 SNPs per individual crab. Additional filtering in Stacks for data present in 75% of individuals and a MAF of 0.05 retained 15,847 SNPS. A final filtering step in PLINK for missing genotypes yielded our full SNP panel of 9,137 loci resulting in a full SNP panel for 241 individual crabs sampled across the 11 sites. Overall observed heterozygosity (*H*_o_) for the full SNP panel (Figure S1A) was similar to that expected (*H*_e_) (0.254 versus 0.256, respectively). Average *H*_o_ for the neutral panel (0.254) was lower than that of the most divergent SNPs (0.290, see below). The global unbiased *F*_ST_ for the full SNP panel was 0.052. Locus‐specific *F*_ST_ values ranged from 0.00001 to 0.551, and heterozygosities ranged from 0.095 to 0.564 for the full SNP panel (Figure S1B). More than half (52%) of the loci exhibited an *F*_ST_ >0.05.

### 3.1 Outlier SNP detection

We utilized three detection methods to obtain our outlier SNP panel. BayeScan detected 398 SNPs putatively under divergent selection (~4.4% of all loci). The nonhierarchical island model based method in Arlequin detected 548 SNPs under selection, while the hierarchical island model based method detected 332 SNPs; 281 loci were common to both of the models implemented in Arlequin. Among all three methods of outlier detection, there were 108 (1.2% of all SNPs) putatively adaptive loci (Figure 3). Outlier loci‐specific *F*_ST_ values ranged from 0.199 to 0.551 with a mean *F*_ST_ of 0.291 relative to 0.043 for neutral SNPs (Figure S2). Our final neutral panel of SNPs was comprised of 8,326 loci after removing any outliers identified by all of the three outlier detection methods.

### 3.2 Population structure

**Figure 4:**
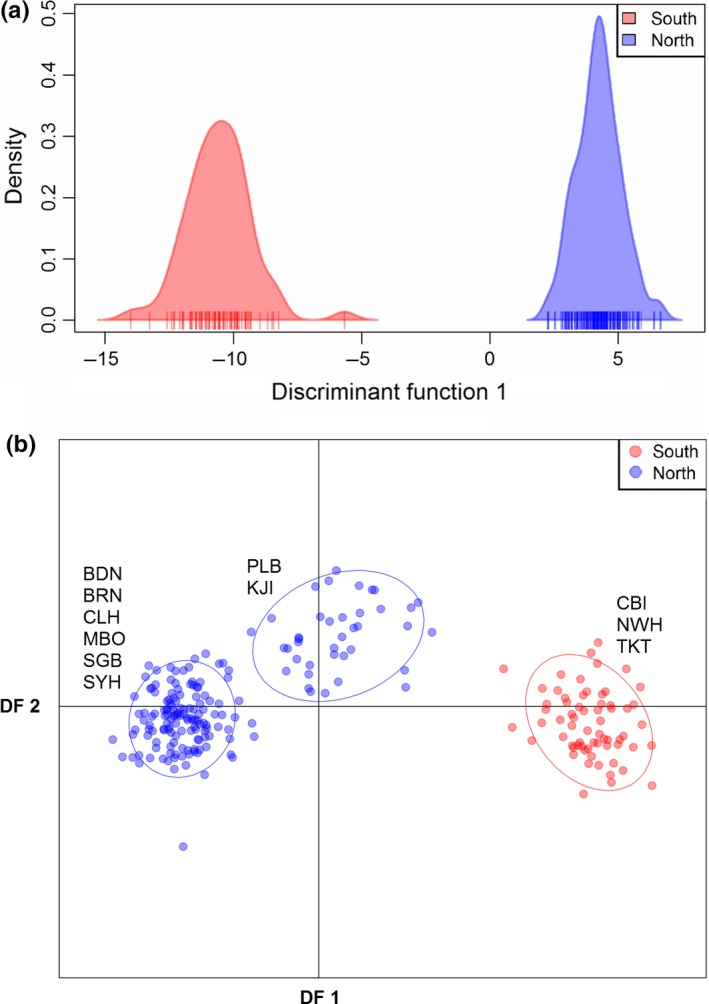
Discriminant analyses of principal components for the (a) neutral SNP panel where *K* = 2, displaying the northern and southern populations along a single discriminant function, and (b) outlier SNP panel where all 11 sites are separated into *K* = 3 clusters spatially distributed along the first two discriminant functions. DAPC on the full SNP panel showed the same results as the neutral panel

DAPC on the full SNP panel indicated the presence of two clusters based on the Bayesian information criterion (Kstat = 1695.06, Figure 4a). The DAPC had a proportion of conserved variance of 0.442 explained by a single discriminant function for the full RAD panel. Both clusters, representing northern and southern groups, had 100% assignment proportions. Similar results were obtained with the neutral SNP panel where again *K* = 2 was best supported (Kstat = 1666.30), with a proportion of conserved variance of 0.779 explained by a single discriminant function and 100% correct assignment for both groups. In contrast, DAPC of outlier SNPs suggested a range of clusters from *K* = 2 to 3 (Kstat = 692.69–693.31, respectively) and had a proportion of conserved variance of 0.925 (Figure 4b). DAPC analyses based on *K* = 2 and *K* = 3 showed the same geographic partitioning as the full and neutral panels, but with KJI and PLB (Cluster 2) intermediate along discriminant function 1 to the pure northern and southern clusters (Clusters 1 and 3; Figure 4b). Principal component analyses on outlier and neutral SNPs revealed the same overall population structure as the DAPC, with clear separation of the northern and southern populations along the first principal component for both the neutral and outlier locus datasets (Figure S3).

**Figure 5:**
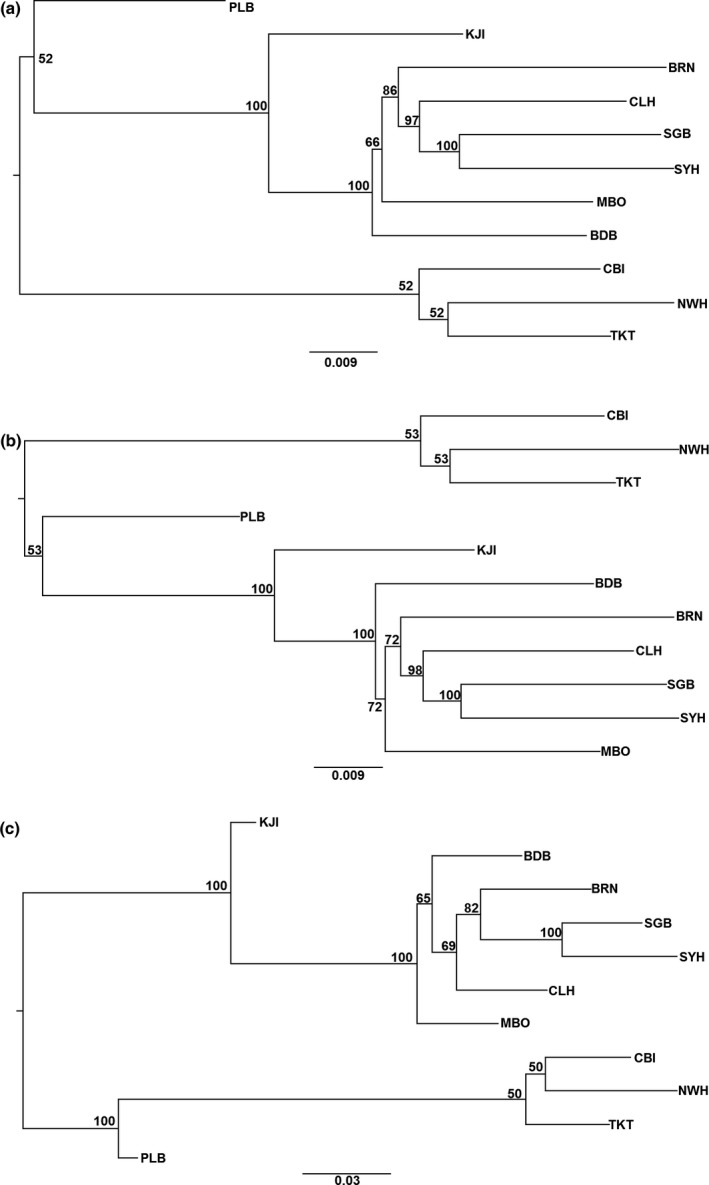
Neighbor‐joining trees based on Cavalli‐Sforza and Edwards (1967) chord distances for three panels of SNPs: (a) the full SNP panel, (b) putatively neutral SNPs, and (c) putatively adaptive SNPs. Values at the nodes represent bootstrap values from 1,000 pseudoreplicates

Neighbor‐joining dendrograms of chord distances showed high support for northern and southern populations for each SNP panel. Bootstrap support for each cluster using the outlier SNP set was 100, with PLB clustering with the southern population. The neutral SNP panel had bootstrap support values of 52–100, but PLB clustered with the northern population in this case (Figures 5 and S4). Bootstrap support for the northern populations was 100 when excluding PLB in all cases. The chord distances for the outlier tree was twice the magnitude of the full or neutral SNP panels.

**Figure 6:**
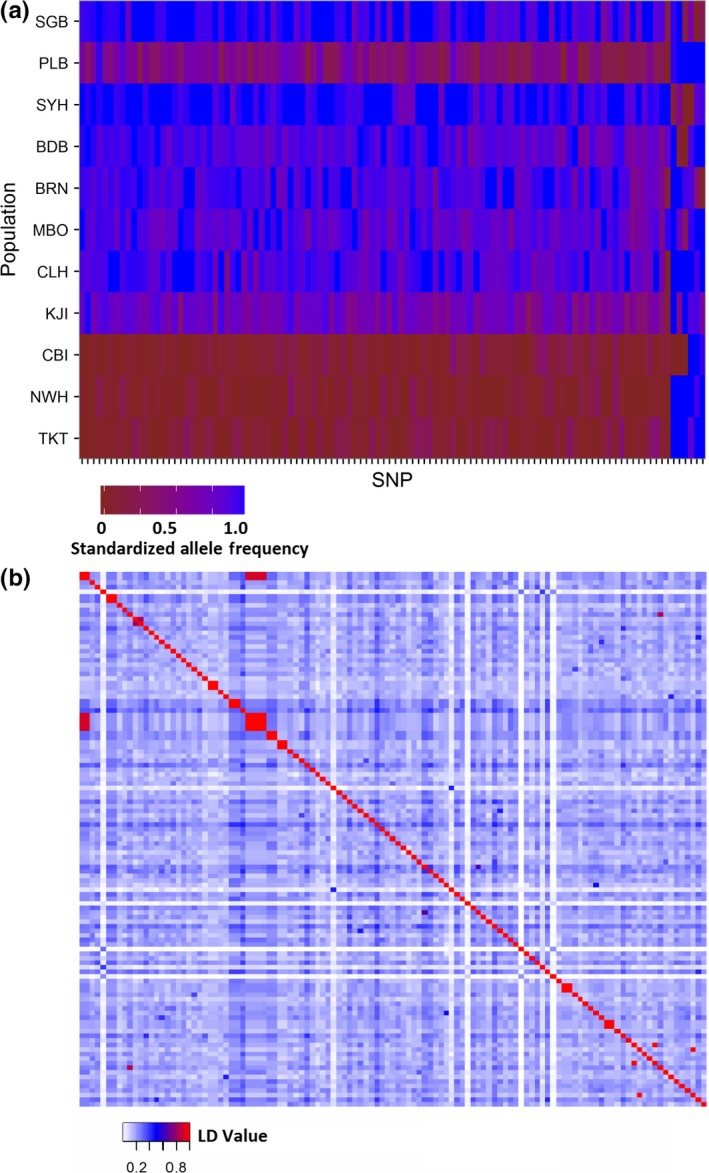
(a) Spatial clines in standardized allele frequency at each outlier locus for the 11 sampling sites sorted by latitude. (b) Heat map of linkage disequilibrium *r* ^2^ values for each outlier locus. Those loci with an *r* ^2^ of 1.0 were located on the same RAD tag, and so only, one locus per RAD tag was retained

Standardized allele frequencies of the outlier loci and neutral loci revealed a clear latitudinal cline overall with a sharp change in allele frequency occurring between CBI and KJI (Figures 6a and S5). Interestingly, PLB shared more alleles with southern sites than northern sites (Figures 6a and S5). Linkage disequilibrium *r* ^2^ values among outliers ranged from 4.75^−07^ to 0.92 following removal of loci located on the same RAD tag with *r* ^2^ values of 1.0 (Figure 6b).

### 3.3 Marker comparisons

**Figure 7:**
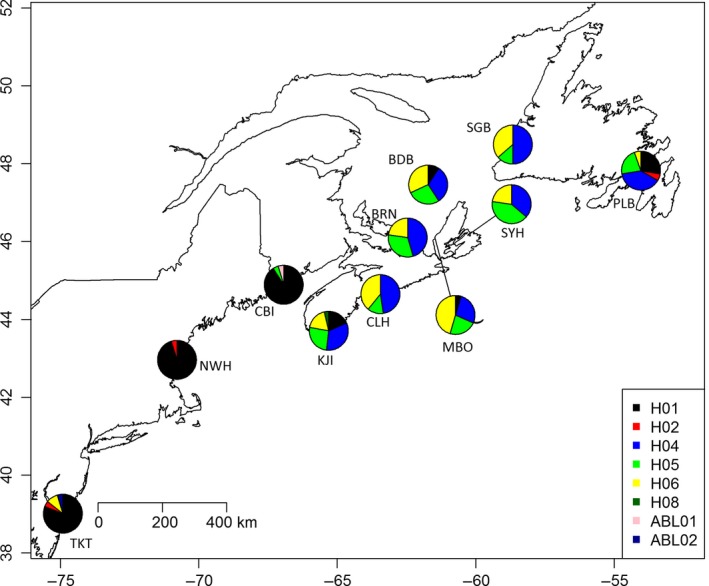
Mitochondrial DNA (COI) haplotypes mapped onto each location, showing a transition in haplotype frequency from the southern green crab population to the northern population starting in Nova Scotia, Canada

**Figure 8:**
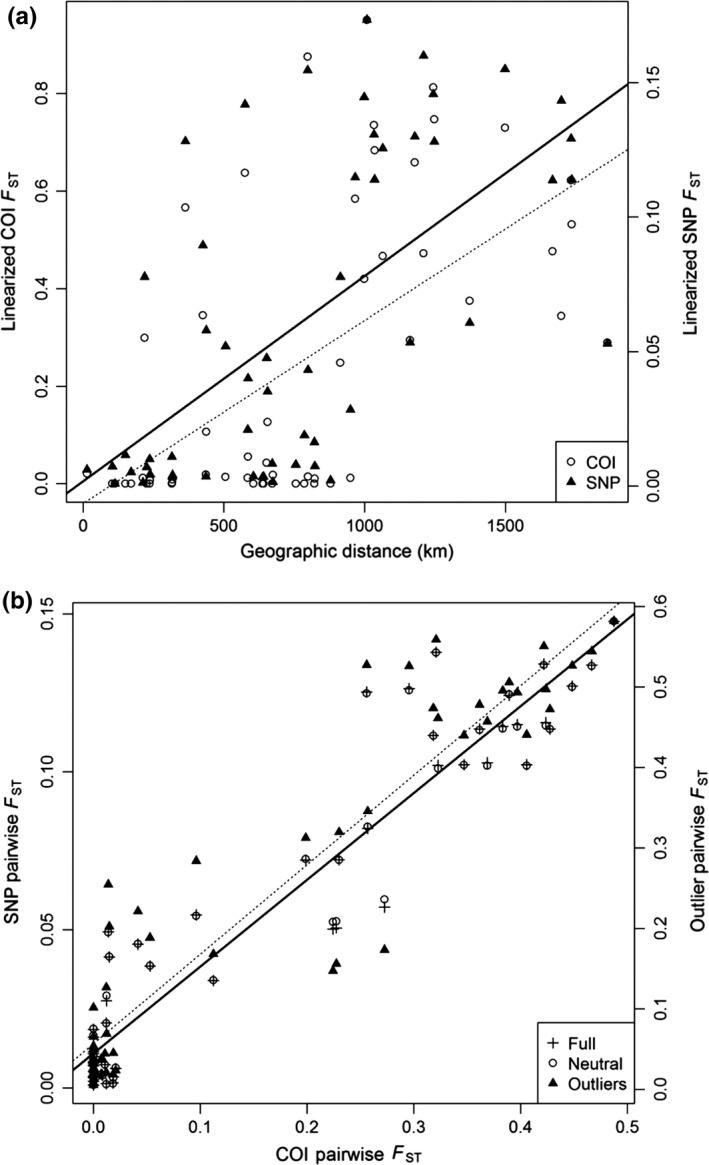
(a) Isolation by distance relationships of linearized pairwise *F*_ST_ values versus least‐cost geographic distances between each site. Correlations were significant for both COI using a Mantel test with 1,000 permutations (*r* = .60, *p* = .008) and the SNP panel (*r* = .624, *p* = .004). The solid line represents the SNP correlation, while the dotted line represents the COI correlation. (b) Comparison of spatial structuring at all three SNP panels and mtDNA data. Mantel tests with 1,000 permutations showed strong (*r* = .93–.95) and significant (*p* < .001) correlations for each dataset. The solid line indicates the full and neutral SNP correlation, while the dotted line indicates the outlier relationship

Haplotype distributions displayed a clear transition from dominance of haplotype H01 in the southern population to dominance of haplotypes H04 to H06 in the northern population (Figure 7). However, approximately 25% of the crabs collected at KJI and PLB were of the southern haplotype. This geographic split mirrors that exhibited by the spatial clustering analysis of the RAD‐seq data above. Pairwise *F*_ST_ values were significantly different between northern and southern populations for all datasets (Table 2, Figure S6). *F*_ST_ values ranged from 0 to 0.49 for the mitochondrial data and from 0.001 to 0.15 for the full SNP panel. Pairwise *F*_ST_ values for the outlier SNPs were similar in range to the COI data, ranging from 0.005 to 0.58, while values for the neutral SNPs were similar to the full SNP panel, ranging from 0.001 to 0.14. Mantel tests on linearized pairwise *F*_ST_ values (Slatkin, 1995) versus least‐cost geographic distances between sample sites showed a significant positive correlation for the full SNP panel (*r* = .624, *p* = .004) and the COI data (*r* = .60, *p* = .008; Figure 8a). Mantel tests comparing pairwise *F*_ST_ values for COI to each SNP panel were highly significant (*r* = .93, outlier SNPs; *r* = .95, neutral/full SNPs, *p* < .001; Figure 8b). AMOVA showed significant genetic differentiation between northern and southern populations when using the neutral SNP panel (*F*_CT_ = 0.058, *p* = .004; Table 3a) and the outlier SNPs (*F*_CT_ = 0. 389, *p* = .001; Table 3b). The outlier SNPs explained substantially more of the variation among groups (38.86% versus 5.76% for the neutral SNPs). Consistent with the SNPs, AMOVA based on the COI variation showed significant genetic differentiation between northern and southern populations (*F*_CT_ = 0.197, *p* = .002; Table 3c), with 19.65% of the variation among groups.

**Table 2:** Pairwise *F*_ST_ values for each population for the COI data (below black diagonal) and the full SNP panel (above black diagonal) ordered by latitude from south to north. Negative *F*_ST_ values were set to zero. Values in bold are significant at *p* < .01, and shaded cells represent north versus south pairwise comparisons

	TKT	NWH	CBI	KJI	CLH	MBO	BRN	SYH	BDB	SGB	PLB
TKT		**0.00353**	**0.0021**	**0.07212**	**0.10226**	**0.13414**	**0.10207**	**0.11152**	**0.11436**	**0.12526**	**0.05022**
NWH	0		**0.00345**	**0.08214**	**0.1135**	**0.14767**	**0.11491**	**0.12421**	**0.12712**	**0.13787**	**0.05717**
CBI	0	0.00264		**0.07215**	**0.10227**	**0.13382**	**0.10289**	**0.11355**	**0.11554**	**0.12629**	**0.05061**
KJI	**0.19868**	**0.25674**	**0.23006**		**0.00732**	**0.02052**	**0.00788**	**0.01139**	**0.01605**	**0.01852**	**0.02758**
CLH	**0.31817**	**0.38925**	**0.36155**	0		**0.00429**	0.00087	0.00151	0.00608	**0.00403**	**0.03393**
MBO	**0.34707**	**0.42742**	**0.40582**	0.01056	0.01821		**0.00518**	**0.00346**	**0.00986**	0.00131	**0.0547**
BRN	**0.38332**	**0.44831**	**0.42356**	0	0	0.02062		**0.00359**	0.00718	**0.00701**	**0.03851**
SYH	**0.42181**	**0.48735**	**0.46673**	0.01194	0.01843	0.00722	0		**0.00824**	**0.00325**	**0.04136**
BDB	**0.32257**	**0.39699**	**0.36872**	0	0	0	0	0		**0.01077**	**0.04542**
SGB	**0.25572**	**0.32073**	**0.29568**	0	0	0.00856	0	0.01187	0		**0.0494**
PLB	**0.22428**	**0.27265**	**0.2275**	0.01207	0.01468	**0.11221**	0.04152	0.09609	0.05281	0.01381	

**Table 3:** Analysis of molecular variance (AMOVA) for the (a) neutral SNP panel, (b) outlier SNP panel, and (c) COI data after categorizing each sampling location into a northern or southern population based on discriminant analysis of principal components

Source of variation	Variance components	Percentage of variation
(a)		
Among groups	70.52	5.76
Among populations within groups	23.30	1.90
Within populations	1129.57	92.33
*F*_ST_ = 0.0767; *F*_SC_ = 0.0202; *F*_CT_ = 0.0576		
(b)		
Among groups	11.51	38.86
Among populations within groups	1.16	3.90
Within populations	16.96	57.24
*F*_ST_ = 0.428; *F*_SC_ = 0.043; *F*_CT_ = 0.389		
(c)		
Among groups	0.28	19.65
Among populations within groups	0.057	3.98
Within populations	1.10	76.37
*F*_ST_ = 0.236; *F*_SC_ = 0.049; *F*_CT_ = 0.197		

## 4 Discussion

The use of genomic approaches to study invasive species demographics and evolutionary history has become increasingly common with high‐throughput sequencing technology, building upon previous studies that have used traditional genetic markers such as mitochondrial DNA and microsatellites (Cristescu, 2015). We used RAD‐seq to generate a panel of genomewide SNPs to examine fine‐scale population structuring in invasive European green crab along the east coast of North America. Our results indicate unusually high levels of genomewide differentiation among invasions consistent with observed differences in northern invasion success. We observed strong evidence for two genetically and geographically distinct populations of green crab spatially segregated in eastern North America corresponding to two independent invasions (Darling et al., 2008, 2014), which show clear latitudinal clines in RAD‐seq‐derived SNP allele frequency. As observed in other species (e.g., Atlantic salmon (*Salmo salar*), Freamo, O'Reilly, Berg, Lien, & Boulding, 2011; Moore et al., 2014; sea scallop (*Placopecten magellanicus*), Van Wyngaarden et al., 2017), our most divergent SNPs (i.e., outliers) explained more of the spatial variance in allele frequency than either background (neutral) SNPs or COI data. Interestingly, genomewide divergence was so prominent that background genomic variation (i.e., neutral SNPs) explained an order of magnitude more of the variation between northern and southern groups than observed in native species in the region (e.g., sea scallop, Van Wyngaarden et al., 2017), and locus‐specific *F*_ST_ values were an order of magnitude larger across the genome than observed in native species [e.g., American lobster, *Homarus americanus*, Benestan et al. (2015) or sea scallop]. This work builds directly on previous studies describing the spatial variation of these two invasions by providing enhanced genomic and geographic resolution of the differences. The extent and magnitude of differentiation across the genome was unexpectedly high for a marine species, which suggests long‐standing and deep divergence exists between the independent invasions which has been maintained in the northern and southern European source populations.

### 4.1 Overall spatial population structure

Our results clearly reveal high levels of genomewide differentiation separating the two invasions into northern and southern groups. This is consistent with the findings of Tepolt and Palumbi (2015) who suggest secondary contact as the dominant structuring force in the introduced range. The presence of two distinct groups of green crab has been consistently reported previously using mtDNA (Roman, 2006), microsatellites (Darling et al., 2014), and RNA‐seq SNPs (Tepolt & Palumbi, 2015). This broad structuring of the two independent invasions in turn mirrors the spatial structuring of the native European range and is likely maintained by environmental differences between the northern and southern regions (Roman, 2006). Our spatial analysis did reveal that two locations (PLB and KJI) appear intermediate between the northern and southern clusters which may be indicative of admixture and hybridization (Darling et al., 2014; Tepolt & Palumbi, 2015). One of these locations, Placentia Bay, is characterized by heavy shipping traffic, and the introduction of a previously admixed population from the Scotian Shelf is likely being maintained at this site (Blakeslee et al., 2010). The other location, Kejimkujik, represents the area where the two invasions (north and south) are coming into contact which appears to be the likely source of observed hybridization (Darling et al., 2014). Previous studies suggest the possible southward movement of this contact zone over time (Darling et al., 2014; Pringle et al., 2011) so the temporal stability of the patterns observed here remains to be seen.

The presence of these two groups was strongly supported by RAD‐seq loci distributed across the genome. Of interest is the fact that the small panel of highly differentiated loci (i.e., outliers, ~1.2% of loci) detected the same level of population structuring as the full panel of SNPs, reinforcing the use of relatively few “diagnostic” markers for fine geographic scale population identification. Similar conclusions were reached by Moore et al. (2014) and Candy et al. (2015) for Atlantic salmon and Pacific eulachon (*Thaleichthys pacificus*), respectively, where outlier SNPs showed the same population structure as neutral SNPs. Observations of similar spatial patterns in both outlier and neutral SNPs supports conclusions of significant genomewide divergence of the groups observed. For instance, both Moore et al. (2014) and Candy et al. (2015) focus on anadromous fish species where gene flow may be low and genomewide divergence likely. Similarly, in green crab, the two independent invasions likely would, at least initially, have low levels of gene flow as they appear to originate from distant locations in Europe (Roman, 2006). Our observations of genomewide divergence support a hypothesis of significant and long‐standing isolation between the two invasions. Ultimately, isolating mechanisms in both the native or introduced range, the temporal stability of these patterns, and the role of hybridization and selection in structuring these two invasions in North America will require further study and is beyond the scope of this work.

### 4.2 Genetic marker comparisons

Previous studies have used COI sequence variation to study green crab population structure and to trace the invasion history (Darling, 2011; Darling et al., 2008, 2014; Roman, 2006). Overall, our SNP data were similar to COI in terms of resolving the current spatial structure of the invasions and suggests that patterns of divergence found in the mitochondria are widespread across the nuclear genome. We identified fewer COI haplotypes relative to previous studies in this system (Darling et al., 2008, 2014) and less evidence of northern haplotypes in the southern population. Nearly 25% of crabs at both KJI and PLB were of the southern haplotype, suggesting anthropogenic introduction from a mixed or hybridized Scotian Shelf population source to PLB (Blakeslee et al., 2010), and secondary contact between northern and southern populations at KJI where the southwards expansion of the more recent invasion met the northern limit of the initial invasion (Darling et al., 2014). Overall, the COI data were equally able to detect isolation by distance and population structuring via pairwise *F*_ST_ values among sampling sites compared to the SNPs. The SNP panel clearly revealed this divergence was widespread across the genome and the outlier SNPs were better able to detect genetic differences between the northern and southern groups explaining significantly greater components of the spatial variance.

### 4.3 Management and conservation implications

The data presented here are consistent with previous descriptions of green crab invasion history (Darling et al., 2014; Tepolt & Palumbi, 2015), suggesting two groups with southern Nova Scotia acting as the (potentially transient) geographic divide between northern and southern populations. This location is consistent with an initial observation by Darling et al. (2014) who documented an admixture zone between northern and southern populations between New Brunswick and Nova Scotia. Knowledge of invasion histories is necessary to predict secondary spread, which ultimately will determine the impact of an invasive species, particularly those where introduction and spread are mediated by anthropogenic vectors (Cristescu, 2015). A better understanding of the population structuring and environmental factors that sustain this structuring within this invasive species will allow us to monitor and potentially predict range expansion, which in turn helps to focus the development of preemptive and targeted monitoring and mitigation strategies in Atlantic Canada. Green crab have the potential to impact commercial shellfish fisheries (e.g., McClenachan et al., 2015) and lead to regional reduction of eelgrass beds (Garbary et al., 2014; Matheson et al., 2016; Neckles, 2015), and so management strategies need to be implemented to minimize their environmental impact. In fact, it may be necessary to consider these crabs as two distinct ecotypes across their invasive range due to their potential adaptations to different thermal regimes and behavioral differences (Rossong et al., 2012) to implement more effective management strategies. Green crab show different thermal tolerances between lineages (Tepolt & Somero, 2014) and adaptive differences in the native range likely facilitated the invasion success and subsequent range expansion in North America (Roman, 2006). However, to date green crab in Atlantic Canada have been treated as a single species and population for risk assessment and habitat suitability modeling (Therriault, Herborg, Locke, & McKindsey, 2008). As evidence suggests there are indeed two ecotypes of green crab in Atlantic Canada, this will necessarily influence future policy and mitigation strategies that account for high and low risk areas for each ecotype that have likely adapted to different temperature regimes. Predictions of habitat suitability and the potential for range expansion would in this case be best evaluated for each ecotype independently.

### 4.4 Summary

The recent green crab range expansion in eastern North America appears to be driven by a second invasion from the northern region of their native range in Europe (Roman, 2006). Our results show that genetic differences between the northern and southern populations of green crab in eastern North America are extensive and genomewide and present strong support for two genetically distinct groups of crabs that were the result of two separate invasions. These crabs may continue to expand northwards and southwards, and potentially hybridize at contact zones or regions high in shipping traffic. The genetic differences observed here are so distinctive that they warrant the division of green crab into two separate ecotypes in the northwest Atlantic, yet the precise latitudinal boundary between ecotypes has yet to be determined and may be transient. Further characterization of the outlier loci based on a whole‐genome sequence and investigation of introgression between the two populations of green crab studied here will facilitate continued monitoring and future exploration.

## Data Accessibility

DNA sequences: All raw COI mtDNA sequence data will be uploaded to NCBI and accession numbers provided. The RAD‐seq genotypes used in this study as well as all distance matrices and geographic least‐cost distances will be deposited in Dryad prior to final acceptance. All corresponding sample codes and locations are in Table 1 and Figure 2. Raw RAD‐seq reads are available from the NCBI Sequence Read Archive BioProject PRJNA377723 at https://www.ncbi.nlm.nih.gov/Traces/study/?acc=SRP102198. COI sequences are available from GenBank (Accession numbers KY800915 ‐ KY801176). Genotype data, including filtered RAD‐seq data and aligned COI sequences are available from the Dryad Digital Repository at http://dx.doi.org/10.5061/dryad.619t0.

## Conflict of Interest

None declared.

## Author Contributions

IRB, R. Beiko, and CDB conceived of the study. JF, KM, CHM, R. Bernier, and CDB collected specimens for use in this study. LCH conducted DNA extraction and RADseq library preparation. NWJ, MVR, RES, PNR, R. Beiko conducted bioinformatics and statistical analyses of the data. All authors aided in the interpretation of the data and initial study design. All authors contributed to writing, revising, and approving the final draft of the manuscript.
