# Supplementary material for: RAD sequencing reveals genomewide divergence between independent invasions of the European green crab (Carcinus maenas) in the Northwest Atlantic
Source: Ecol Evol. 2017 Mar 14;7(8):2513–24. doi: 10.1002/ece3.2872 (PMC5395438; doi:10.1002/ece3.2872)
Supplement: Supplementary file 1 [file ECE3-7-2513-s001.doc]

**Supplementary Figures**


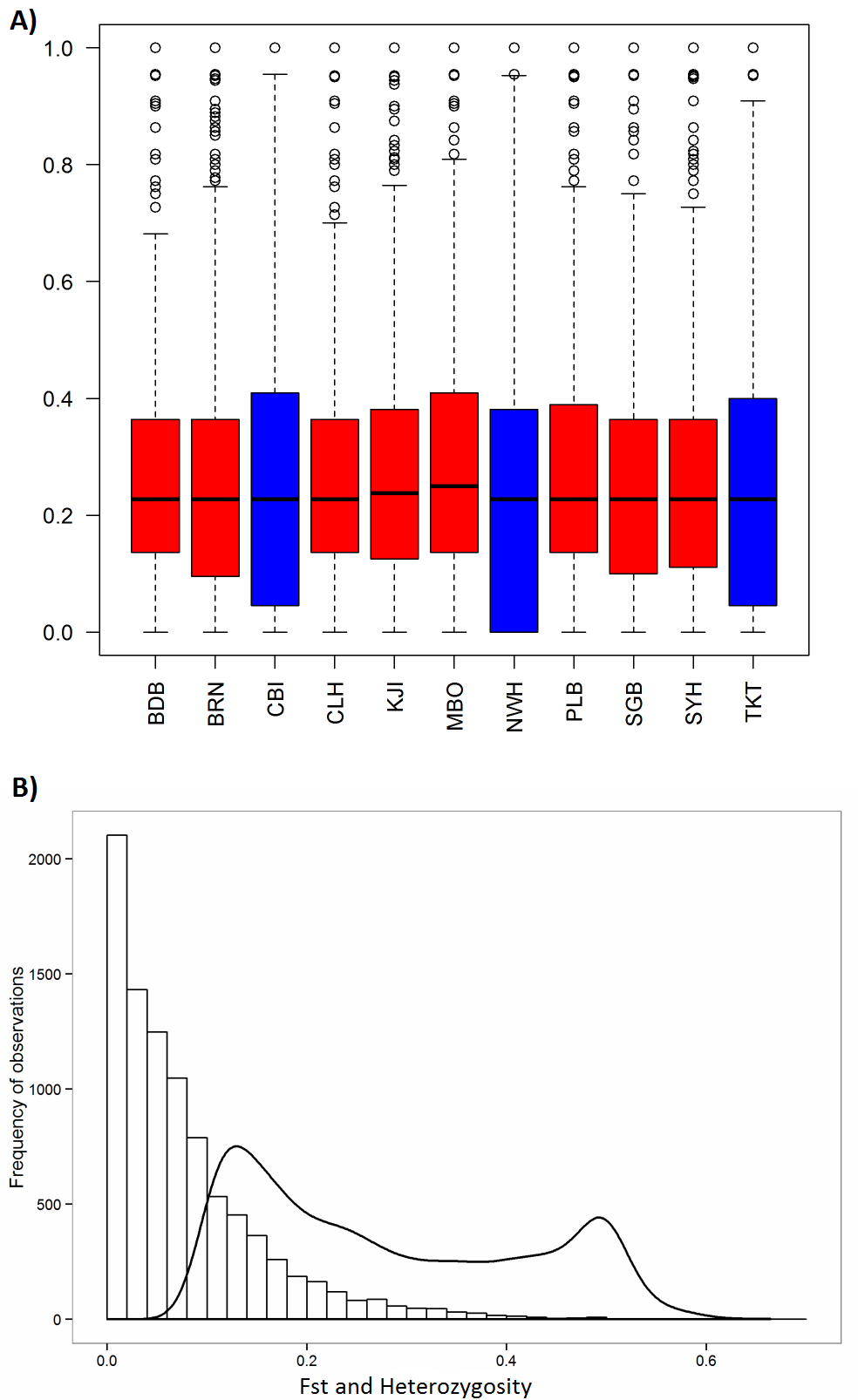


Figure S1. A) Box plot of observed heterozygosity for the full SNP panel for all 11 sampling sites. Red boxes indicate northern sites and blue boxes indicate southern sites. B) Locus-specific *F*_ST_ values (bars) and heterozygosity (line) frequencies.


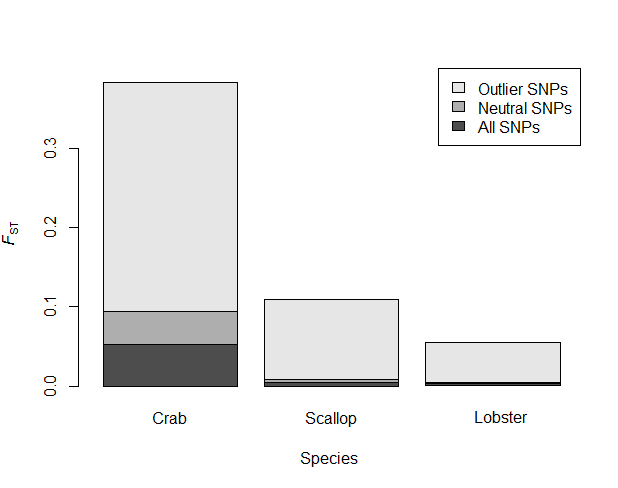


Figure S2. Average locus-specific *F*_ST_ values for outlier, neutral, and full SNP panels from three marine species found in the northwest Atlantic. Data for sea scallop (*Placopecten magellanicus*) from Van Wyngaarden et al. (2016), American lobster (*Homarus americanus*) from Benestan et al. (2015), and green crab (*Carcinus maenas*) from the present study.


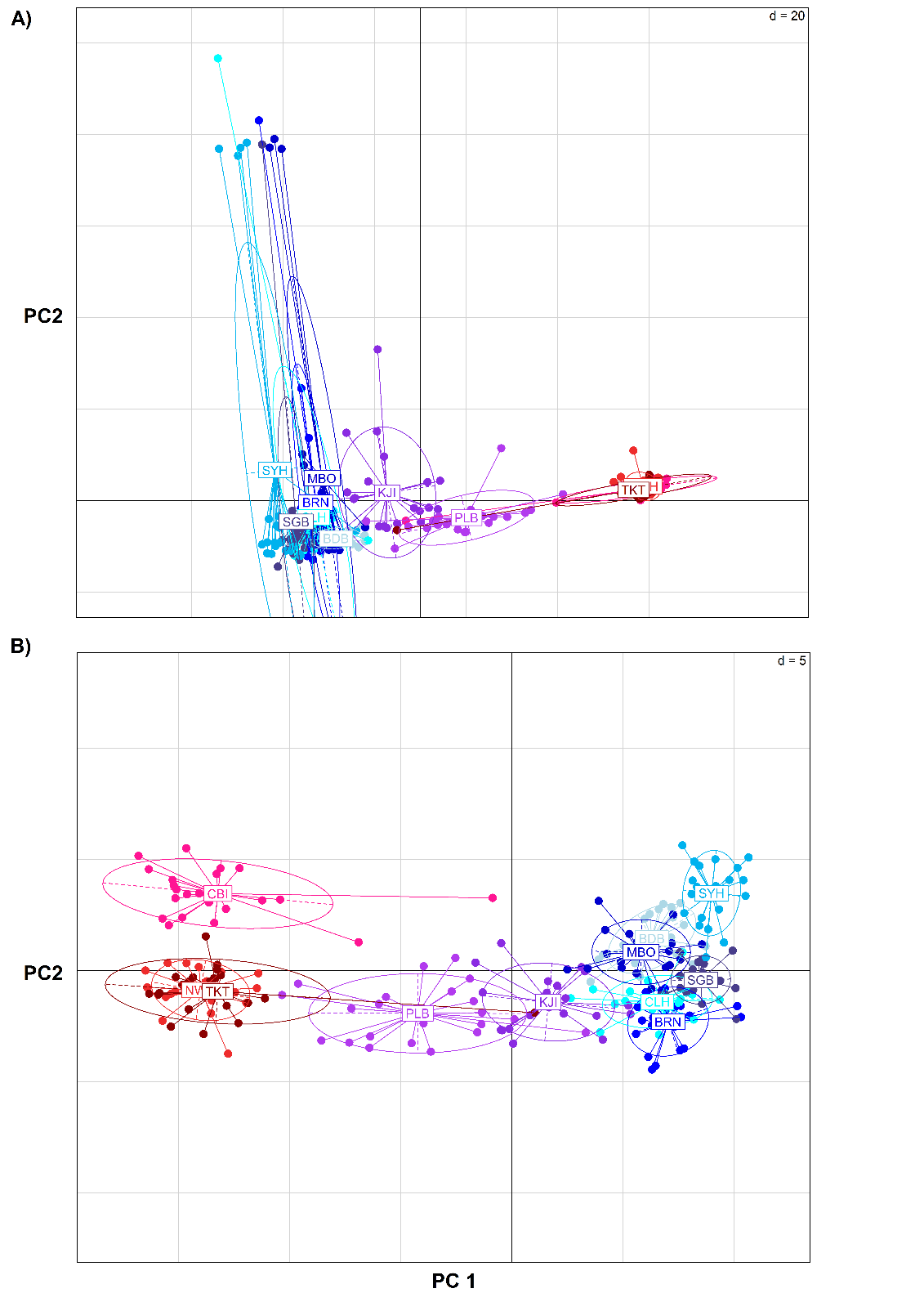


Figure S3. Principal component analysis of A) neutral loci genotypes and B) outlier loci genotypes by population. Division of northern and southern ecotypes is evident along the first principal component axis, and KJI and PLB represent admixed populations as also shown by a discriminant analysis of principal components (DAPC).


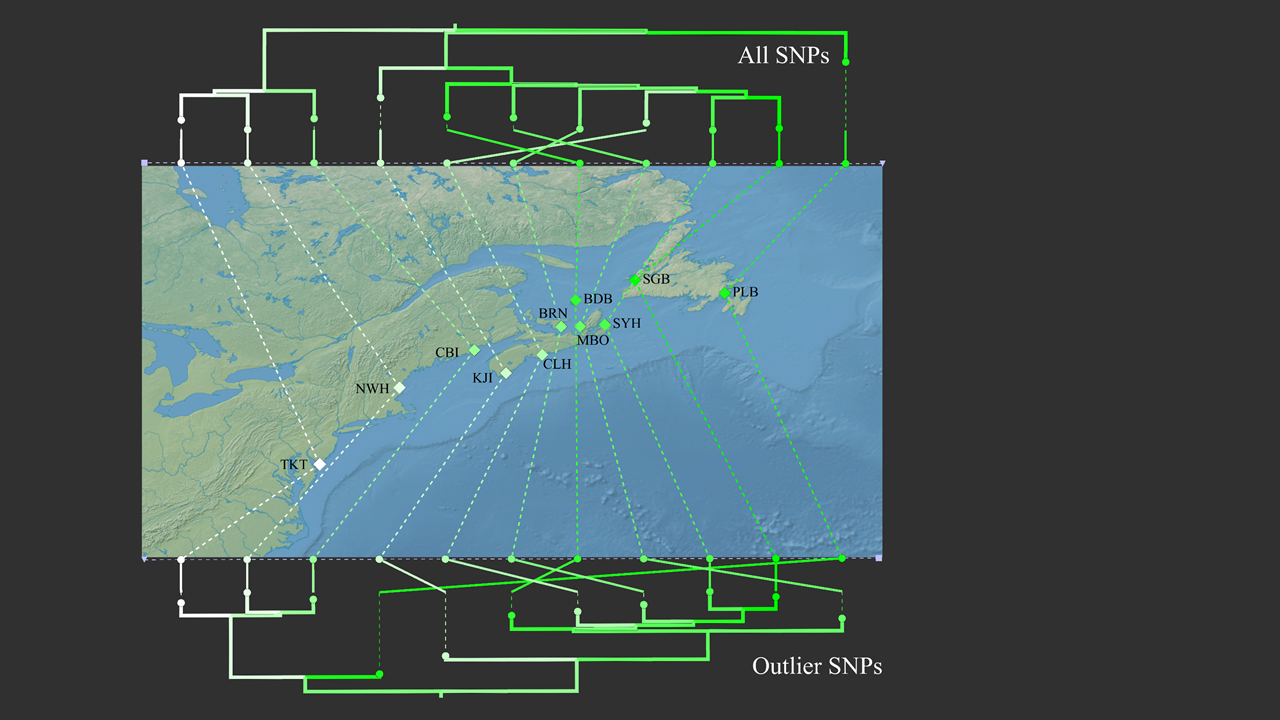


Figure S4. Clustering of locations based on Cavalli-Sforza and Edwards (1967). Dashed lines connect locations to their corresponding positions in the NJ trees based on all SNPs (top) or outlier SNPs only (bottom). Locations are coloured according to latitude, from white (southernmost location) to green (northernmost location).


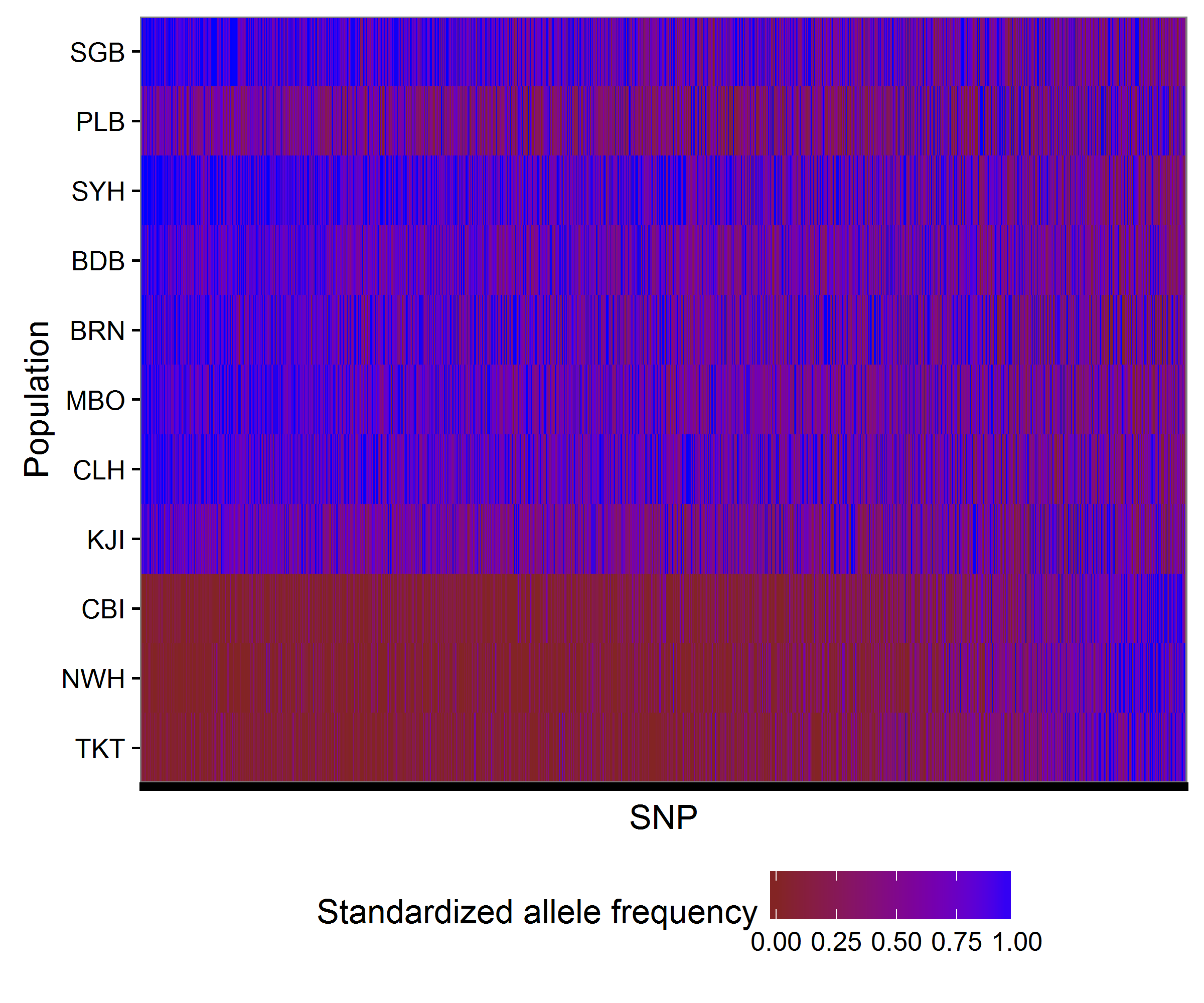


Figure S5. Heatmap of standardized allele frequencies of 8326 putatively neutral SNPs. Sites on the y-axis are arranged by latitude, and a clear transition in allele frequency occurs with latitude.


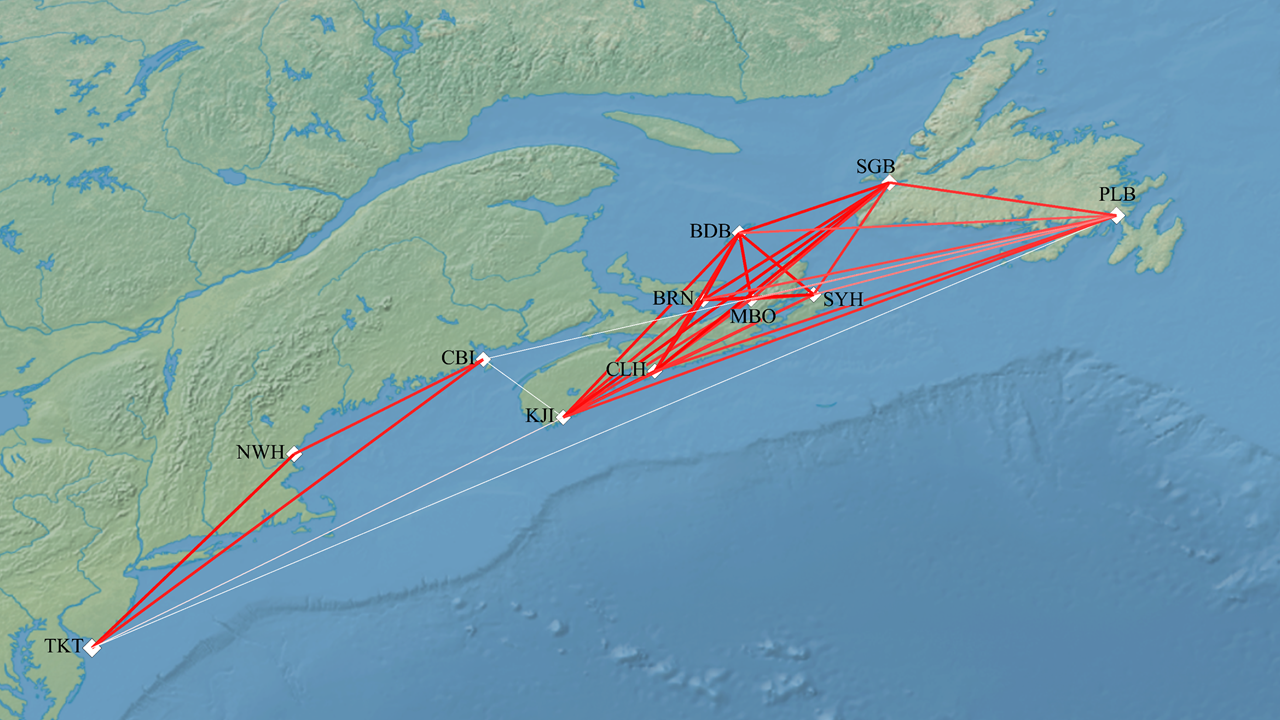


Figure S6. Visual representation of *F*_ST_ distances between sites with a maximum *F*_ST_ score of 0.25. Line thickness and colour is proportional to *F*_ST_ distance, with thickest red lines corresponding to a score of 0, and thin white lines corresponding to a score of 0.25.
